# Genomic characterization and infectivity of a novel SARS-like coronavirus in Chinese bats

**DOI:** 10.1038/s41426-018-0155-5

**Published:** 2018-09-12

**Authors:** Dan Hu, Changqiang Zhu, Lele Ai, Ting He, Yi Wang, Fuqiang Ye, Lu Yang, Chenxi Ding, Xuhui Zhu, Ruicheng Lv, Jin Zhu, Bachar Hassan, Youjun Feng, Weilong Tan, Changjun Wang

**Affiliations:** Aff1 Department of Epidemiology, College of Preventive MedicineThird Military Medical University 400038 Chongqing China; Aff2 Department of EpidemiologyResearch Institute for Medicine of Nanjing Command 210002 Nanjing China; Aff3 Jiangsu Institute of Parasitic Diseases 214064 Wuxi Jiangsu Province P.R. China; Aff4 Stony Brook University 11794 Stony Brook USA; Aff5 Department of Pathogen Biology & Microbiology and Department of General Intensive Care Unit of the Second Affiliated Hospital, Zhejiang University School of Medicine 310058 Hangzhou Zhejiang China

## Abstract

SARS coronavirus (SARS-CoV), the causative agent of the large SARS outbreak in 2003, originated in bats. Many SARS-like coronaviruses (SL-CoVs) have been detected in bats, particularly those that reside in China, Europe, and Africa. To further understand the evolutionary relationship between SARS-CoV and its reservoirs, 334 bats were collected from Zhoushan city, Zhejiang province, China, between 2015 and 2017. PCR amplification of the conserved coronaviral protein RdRp detected coronaviruses in 26.65% of bats belonging to this region, and this number was influenced by seasonal changes. Full genomic analyses of the two new SL-CoVs from Zhoushan (ZXC21 and ZC45) showed that their genomes were 29,732 nucleotides (nt) and 29,802 nt in length, respectively, with 13 open reading frames (ORFs). These results revealed 81% shared nucleotide identity with human/civet SARS CoVs, which was more distant than that observed previously for bat SL-CoVs in China. Importantly, using pathogenic tests, we found that the virus can reproduce and cause disease in suckling rats, and further studies showed that the virus-like particles can be observed in the brains of suckling rats by electron microscopy. Thus, this study increased our understanding of the genetic diversity of the SL-CoVs carried by bats and also provided a new perspective to study the possibility of cross-species transmission of SL-CoVs using suckling rats as an animal model.

## Introduction

Coronaviruses (CoVs) are a family of RNA viruses belonging to the *Coronaviridae* family and the *Coronavirinae* subfamily and are the largest group of positive-sense single-stranded RNA viruses. From an academic perspective, CoV can be divided into four genera, namely *Alphacoronaviruses*, *Betacoronaviruses*, *Gammacoronaviruses,* and *Deltacoronaviruses*. The alphacoronaviruses and betacoronaviruses are usually found in mammals, while the gammacoronaviruses and deltacoronaviruses are mainly associated with birds^1,2^. SARS-CoV is the causative agent of the severe acute respiratory syndrome (SARS) outbreak that occurred in 2002–2003. This SARS outbreak was the first human pandemic to break out since the beginning of the 21st century, and it resulted in nearly 8000 cases of infection and 800 deaths worldwide^3,4^. SARS-CoV belongs to the *Betacoronavirus* genus, and its genomic sequence exhibits low levels of similarity with the previously identified human CoVs-OC43 and 229E. Thus, we hypothesized that SARS-CoV underwent a long and independent evolutionary process. The SARS-CoV genome usually encodes four structural proteins: the spike protein (S), envelope protein (E), membrane protein (M), and nucleocapsid protein (N). Among them, the S protein is a trimeric, cell-surface glycoprotein that consists of two subunits (S1 and S2), whereas the S1 subunit is responsible for receptor binding. Variations in the S protein, to a large extent, are responsible for the tissue tropism and host ranges of different CoVs^5,6^.

The origin of SARS-CoV has always been a focus of research. Palm civets were initially considered the natural reservoir of SARS-CoV due to the isolation of several strains of SARS-CoV from palm civets that were traded in the wet markets of the Guangdong province of China in 2003^7^. However, subsequent studies showed that the virus was detected only in palm civets of market origin that were tested prior to culling, but not in those tested later; palm civets captured from the wild also tested negative for the virus. This finding suggested that palm civets served only as an intermediate reservoir and are therefore not a natural reservoir for SARS-CoV^8,9^. Recently, bats have captured our attention due to their ability to act as natural reservoirs for a wide variety of viruses, including many important zoonotic viruses that are associated with several severe forms of emerging infectious diseases, such as Ebola virus, Nipah virus, Hendra virus, and Marburg virus^10,11^. In 2005, teams from Hong Kong and Mainland China almost simultaneously discovered the presence of SL-CoVs in wild Chinese horseshoe bats (*Rhinolophus sinicus*) from China. These findings suggested that the bats were the natural hosts of SARS-CoV^12^. Notably, during longitudinal surveillance of the *Rhinolophus sinicus* colony in the Yunnan Province of China over the past few years, a Chinese research team successfully isolated a live SL-CoV sample from Vero E6 cells that were incubated in the bat feces in 2013^13^. The isolated virus showed more than 95% genome sequence identity with human and civet SARS-CoVs. Further studies on these indicated that the SL-CoV from bats may directly infect humans and does not require an intermediate host. SL-CoV, similar to SARS-CoVs, possesses the ability to infiltrate cells using its S protein to combine with angiotensin-converting enzyme 2 (ACE2) receptors^14^. This observation indicated that SARS-CoV originated from Chinese horseshoe bats and that SL-CoV isolated from bats therefore poses a potential threat to humans.

In recent years, many novel SL-CoVs have been identified in a variety of bat species throughout the world, including Asia, Europe, Africa, and America. Most SL-CoVs were discovered in *rhinolophids* from China, Slovenia, Bulgaria, and Italy^15–17^, while novel beta-coronaviruses related to SARS-CoV have been detected in *Hipposideros* and *Chaerophon* species from Kenya and Nigeria^18,19^. However, analysis of the RNA-dependent RNA polymerase (RdRp) amino acid sequence showed that the genomic sequences of these bat SL-CoV samples obtained from different parts of the world shared 80–90% identity among themselves and exhibited 87–92% identity with the SARS-CoVs extracted from human or civet sources^20,21^. These findings indicated that SARS-CoV likely evolved in bats over longer periods of time. Previous research conducted by our group revealed that bats found in Southeast China have high carrying capacities for SL-CoVs^22^. After conducting an epidemiological survey on the bats carrying CoVs, two novel SL-CoVs were identified in the *Rhinolophus pusillus* specimens from Zhoushan city, Zhejiang Province, China; subsequently, a rat infection model was utilized to assess the cross-species transmission potential of the viruses.

## Results

### Sampling

Between 2015 and 2017, 334 bats were sampled from Zhoushan, China. These bats belonged to the species *Rhinolophus pusillus* as determined by the sequences of the mitochondrial cytochrome *b* gene in their muscle tissues^23^. All 334 bat samples were screened for CoV RNA using a pan-coronavirus reverse transcription (RT)-PCR assay. The overall prevalence of the virus was 26.65% (89/334, bats; Table 1). Additionally, a higher prevalence was observed in samples collected in July (66.7% in 2015) than in those collected in October (21% in 2016) or February (13% in 2017). A phylogenetic tree was constructed according to the 440-bp RdRp partial sequences, and the positive samples were classified into *Alphacoronaviruses* and *Betacoronaviruses*. As shown in Fig. S1, 89 amplicons were grouped into five clades with 66–100% nucleotide identities between them, and they shared 94–100% identities with the viruses that were extracted from Hong Kong, Guangdong, and Hainan in China as well as those from Spain.

**Table:** Summary of the bat-CoVs detection in bats from the Zhejiang province of China

Time	Locus	Sample number	Bat species	CoV positive
July, 15	Dinghai, Zhoushan city (ZXC)	45	*Rhinolophus sinicus*	66.7% (30/45)
January, 16	Dinghai, Zhoushan city (Z2)	120	*Rhinolophus sinicus*	25% (30/120)
October, 16	Daishan, Zhoushan city (DXC)	84	*Rhinolophus sinicus*	21% (18/84)
February, 17	Dinghai, Zhoushan city (ZC)	85	*Rhinolophus sinicus*	13% (11/85)
Total		334		26.65 (89/334)

### Full genomic sequence comparison and recombination analyses

**Fig. 1:**
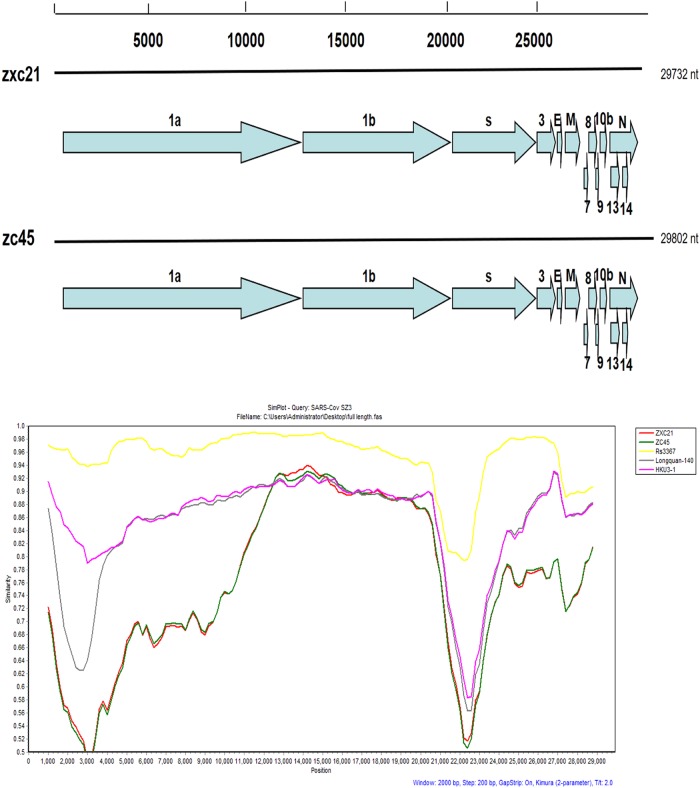
A gene map of the two novel SL-CoVs and the recombination analysis of novel SL-CoVs with other SL- CoVs. Similarity plots were conducted with SARS CoV SZ3 as the query and bat SL-CoVs, including Rs3367, Longquan-140, and HKU3-1, as potential parental sequences. The analysis was performed using the Kimura model, with a window size of 2000 base pairs and a step size of 200 base pairs

To further explore the evolution of SL-CoV from Zhoushan, two complete genomic sequences of the representative bat-derived CoVs were generated by sequencing several overlapping amplicons. Specifically, sequences were generated from the following samples: SL-CoV ZXC21 (MG772934) bat that was extracted from a sample procured in July 2015, and SL-CoV ZC45 (MG772933) bat that was extracted from a sample procured in February 2017. The full genomes of ZXC21 and ZC45 consisted of 29,732 nt and 29,802 nt, respectively. The genomic organization in both cases was similar to that of the most well-known bat-SL-CoVs. Using the RDP program, the potential recombinant events between ZXC21, ZC45 and other representative strains of 13 human/civet and bat SARS-like CoVs were initially predicted. The results did not identify any potential recombination events. The genomic sequence similarity among the five bat-SL-CoVs and the SARS-CoV SZ3 strain was examined by Simplot analysis (Fig. 1). The results showed that the genomes had 38.9% GC content and had 13 open reading frames (ORFs) similar to the HKU3-1 strain. The two new bat SL-CoVs shared 97% genomic sequence identity among themselves. The overall nucleotide sequence identity of these two genomes with civet SARS-CoV (SZ3 strain) was 81%, which was lower than the previously reported observations associated with bat SL-CoVs collected from China (88–92%). From homology analyses of different ORFs, ORF8 fragments showed the lowest homology with the reported SL-CoV homology data^24^, presenting a shared identity of only 60% with its closest relatives.

**Fig. 2:**
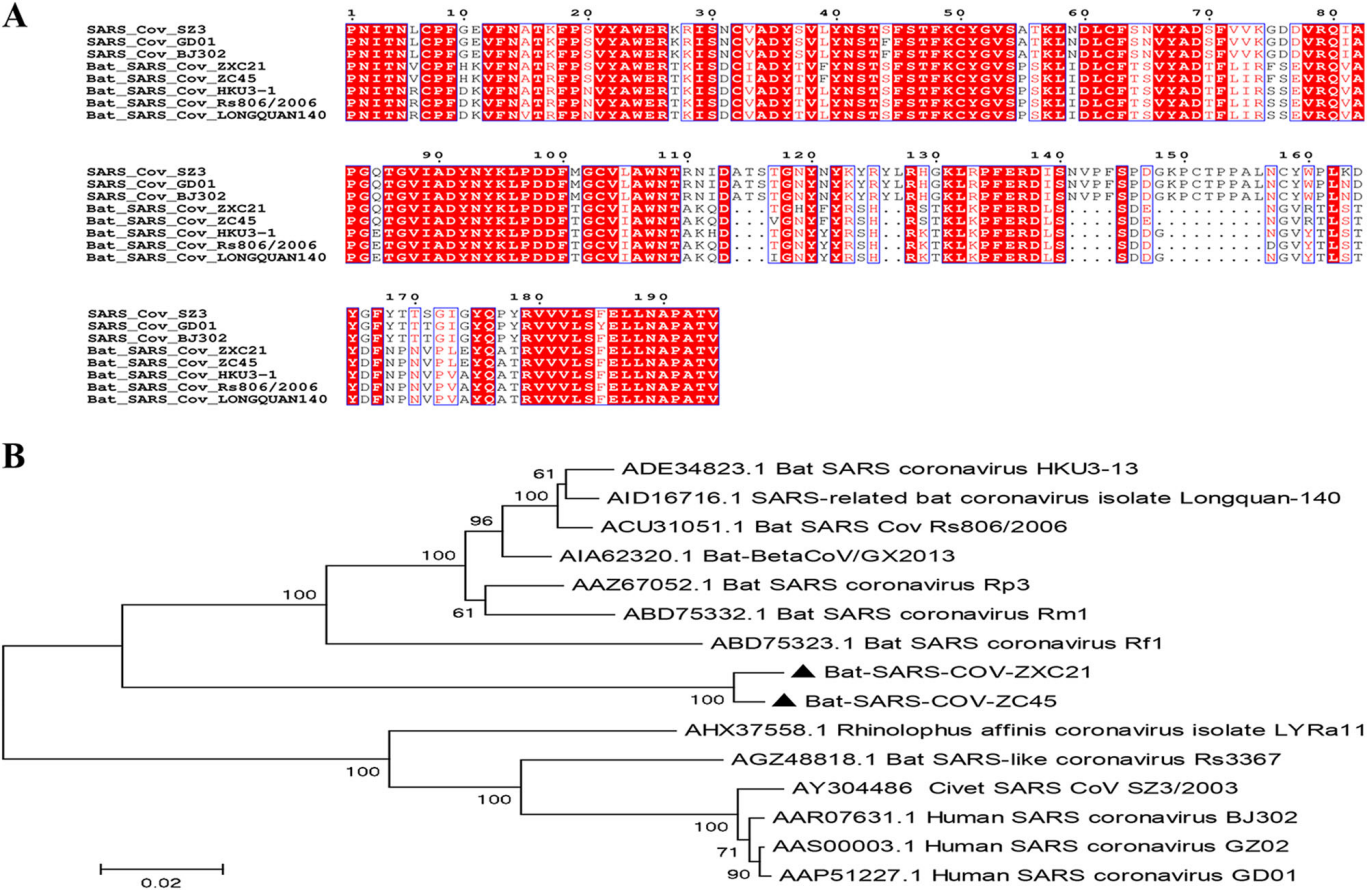
Characterization of S1 domains of the SARS CoV and SL-CoVs. **a** Amino acid sequence comparison of the S1 subunit. The receptor-binding domain (aa 318–510) of SARS-CoV. **b** A phylogenetic analysis of the entire S1 amino acid sequences based on the neighbor-joining method. The SARS-CoV-GD01, BJ302, and GZ02 strains were isolated from patients of the SARS outbreak in 2003. The SARS-CoV SZ3 was identified from civets in 2003. Other bat-SL-CoVs were identified from bats in China.The sequences of SL-CoVs in this study are marked as filled triangles

The S protein is responsible for the entry of the virus and is functionally divided into two domains, S1 and S2. The bat SL-CoV Rs3367 is the most closely related virus to the human SARS-CoV and has 89.9% amino acid sequence identity to the SARS-CoV with respect to the whole spike protein. Comparatively speaking, the S proteins of ZXC21 and ZC45 identified in this study were slightly more different than their counterpart in SARS-CoV, which showed 77% identity at the amino acid level. Phylogenetic analyses based on the S protein suggested that the S proteins of ZXC21 and ZC45 represented a separate clade related to the lineage B CoVs (Fig. 2b). The highest amino acid sequence identity shared with the Rs806 strain was only 83%. Like other bat-SL-CoVs, the S1 domain of the bat SARS-like CoVs exhibited a very low nucleotide similarity with SARS CoV, and there are several key deletions and mutations in most of the variable regions within the receptor-binding domain (RBD) (Fig. 2a).

### Rat infection and virus detection test

**Fig. 3:**
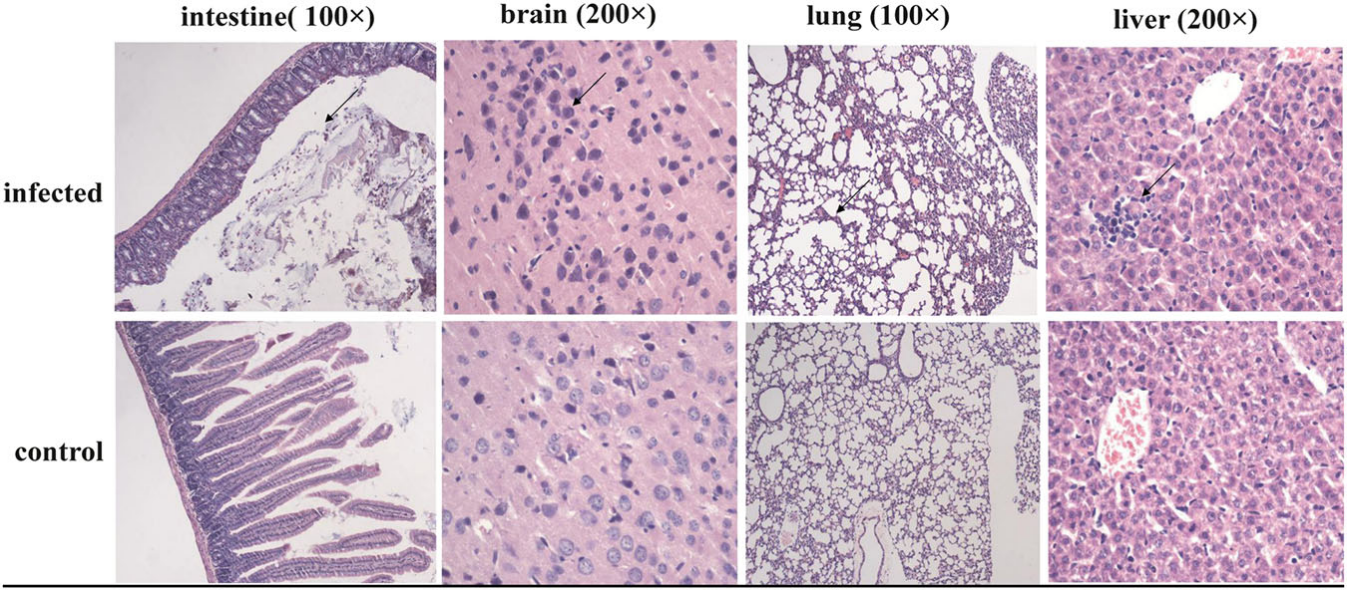
Light microscopy observations of rat tissues infected with bat-SL-CoVs: Sectioned brain, intestine, lung and liver tissues were sampled from rats infected with bat-SL-CoV ZC45

Despite the failed isolation of the infectious virus from PCR-positive samples in Vero E6 cells, we attempted to isolate the virus from suckling rats by infecting them with tissue samples that were positive for the coronavirus. After 15 days, pathological analysis showed that the tissues and organs of the infected rats exhibited varying degrees of inflammation, and the inflammatory reaction in the brain tissues was most evident. Of the ten suckling rats, four showed clinical symptoms, including drowsiness, slow action, and mental depression. The new suckling rats infected with the diseased brain tissue still had irregular onset, whereas five of the 11 suckling rats in one nest had clinical symptoms. Numerous apoptotic neurons were seen in the focal areas of the brain tissue, and the chromatin in the nuclei was condensed and unclear. The lung tissues were well structured, but the alveolar cavities were partly fused together and showed clear signs of mild emphysema. Intestinal tissue analysis showed a loss in the structure of the intestinal mucosa; the mucous membranes were thin, the crypts were shallow, the intrinsic glands were reduced, and the stroma showed a dispersed inflammatory infiltrate (Fig. 3). Subsequently, the viral load of different tissues was detected by quantitative PCR, and the viral loads of the lung tissues remained the highest, showing approximately 10^4^ viral genome copies per 1 μl of tissue suspension (data not shown).

**Fig. 4:**
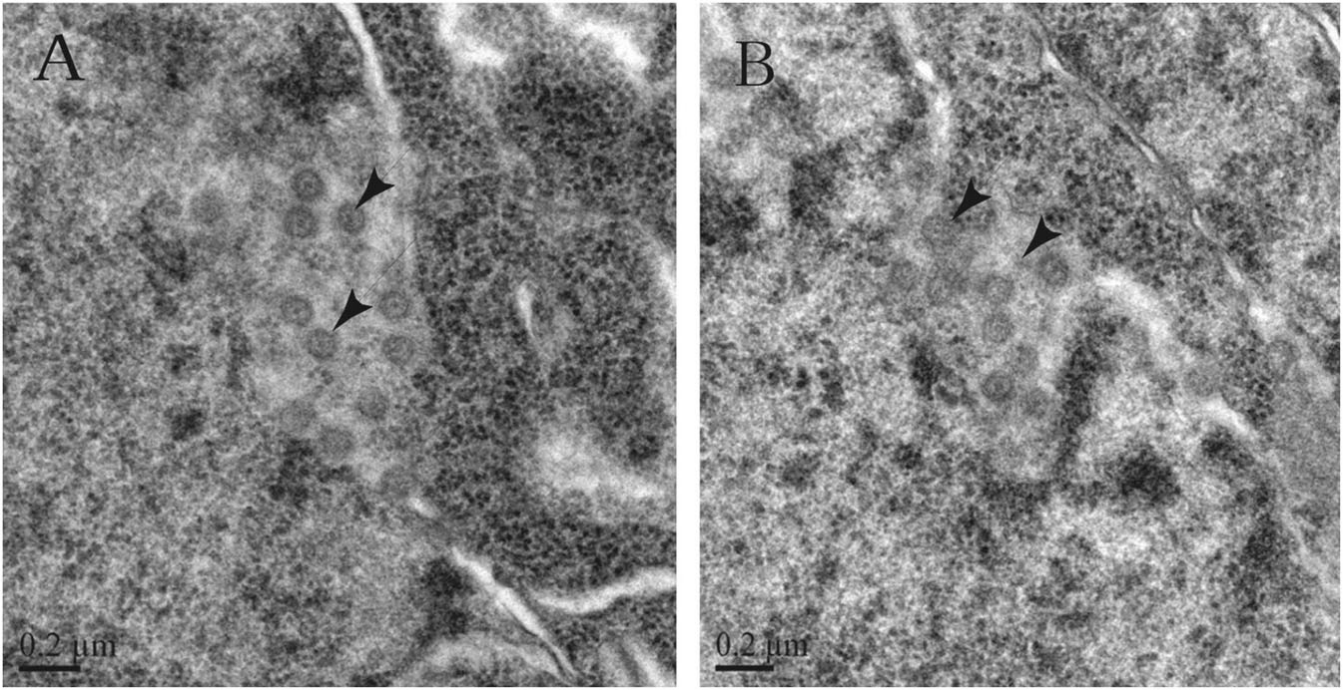
Transmission electron micrographs of infected rat brain tissues. **a, b** CoV-like particles are considered SL-CoVs ZC45 in different locations of the infected rat brain tissues

Suspected viral particles were observed in the nuclei of denatured neurons in the brain tissues of the rats using transmission electron microscopy (TEM). These viral particles presented the typical coronavirus morphology and were approximately 100 nm in size with apparent surface spikes (Fig. 4). Simultaneously, various viral RT-PCR tests were conducted on the tissues to detect viral particles. The tissues were tested for the presence of viral particles associated with a wide variety of viruses, such as CoVs, henipaviruses, respiroviruses, avulaviruses, rubulaviruses, and the influenza-A virus of the *Orthomyxoviridae* family, using previously published methods^25,26^. The test results revealed that the tissues were positive only for CoV.

### Analysis of the N protein antigen and western blotting

**Fig. 5:**
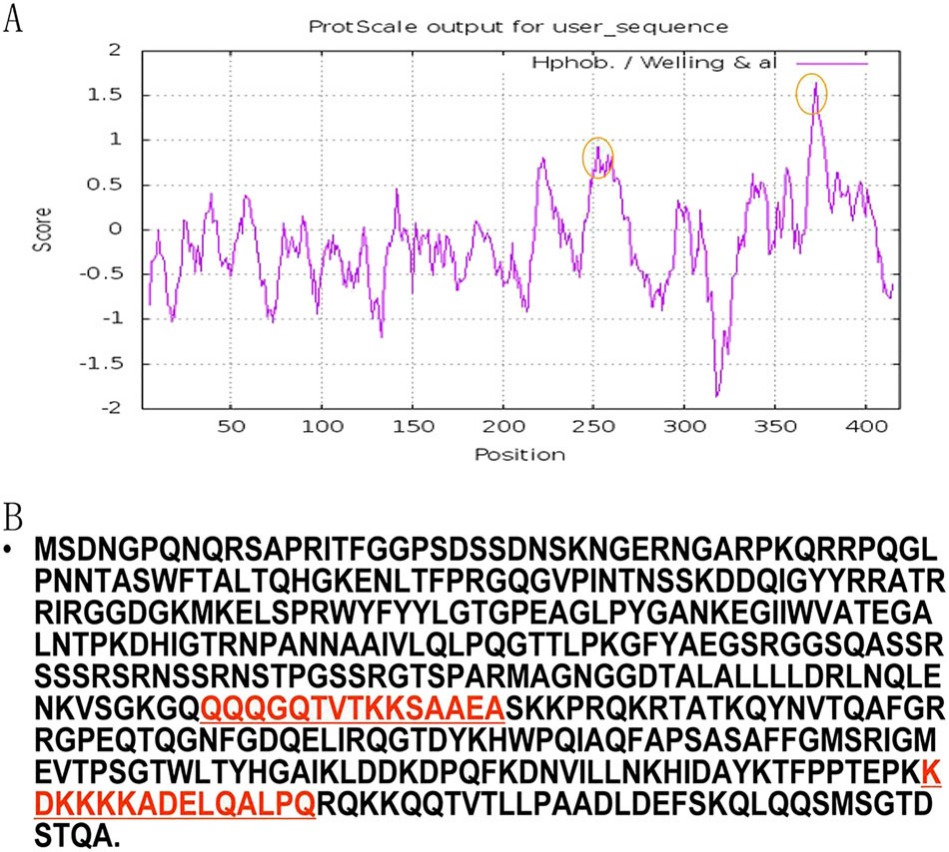
Prediction of the antigenicity of the bat SL-CoV N protein. **a** The predicted antigenicity for the N protein. **b** Amino acid sequence of the N protein. The high antigenicity portion is indicated in the red circle. The two synthesized polypeptides are indicated in red

Similar to other CoVs, the nucleocapsid protein is one of the core components of the SARS-CoV. The N protein is one of the most predominantly expressed proteins during the early stages of SARS-CoV infection and has been an attractive diagnostic tool due to the initiation of strong immune response against it. Evolutionary analyses have shown that the homology between the N protein and its counterparts in the well-known SARS-CoV and bat SL-CoV ranged from 89 to 91%. The antigenic analysis was based on the amino acid sequence of the N protein (Fig. 5), and the results suggested that the two alternative antigenic peptides, including KHD2016288-1:KDKKKKADELQALPQ and KHD2016288-2:QQQGQTVTKKSAAEA, were selected for peptide synthesis

**Fig. 6:**
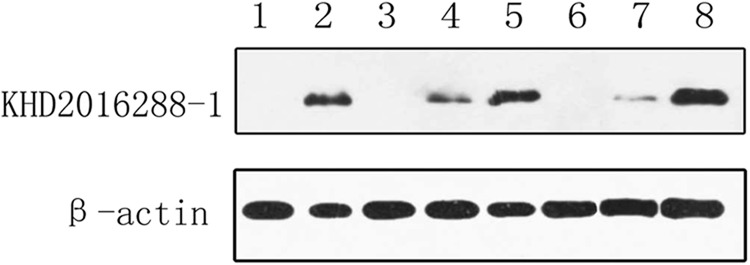
Detection of N protein expression in infected rat tissues by western blotting. Proteins from the following tissues were analyzed: rat brain from the control specimen (lane 1), intestinal tissue from bat ZC45 (lane 2), intestinal tissue from the infected rat (lane 3,6), lung tissue from the infected rat (lane 4,7), and brain tissue from the infected rat (lane 5,8)

To further characterize the antigenic reactivity of the virus in infected murine tissues with ZC45-specific antibodies compared to that of ZC45, polyclonal antibodies against the polypeptides (KHD2016288-1:KDKKKKADELQALPQ) derived from ZC45 N proteins were generated and then subjected to western blotting analysis (Fig. 6). The anti-polypeptides were derived from the ZC45 N protein antibodies from six different sources of N proteins (50 kDa), including the intestinal tissues, brain tissues and lung tissues of infected rats.The results indicated that the polypeptide antigen was synthesized correctly, and the polyclonal antibodies produced against this polypeptide could react with the N proteins of the bat SL-CoV. The polyclonal antibodies reacted specifically with the infected rat tissues, but not with the rat tissues derived from the control specimens. These results indicated that the virus can circulate and proliferate in infected rats.

## Discussion

Since the first report on the origin of SL-CoVs from bats in 2005, CoVs have been found in ten different bat species within six families from more than ten countries, including China, Africa, and Europe^21,27^. Our 2-year longitudinal surveillance of bats in Zhoushan indicated that all 334 bats that were collected belonged to the species *Rhinolophus sinicus*, suggesting that it was the dominant bat species found in our study and has been shown to be the natural reservoir of SARS-CoV. Nested PCR amplification of the conserved region of RdRp showed that the CoV carrying rate associated with this species of bat was much higher than that reported previously^28,29^. At the same time, the summer carrying rate was higher than that associated with the other seasons due to the influence of seasonal distribution. In this region, there were two clades of *Alphacoronaviruses* and three clades of *Betacoronaviruses* identified, indicating that a wide variety of CoVs circulate in the bats of the Zhoushan area, and these CoVs were the most widely transmitted in the bat colonies found in this region.

To explore the possibility of CoV transmission from bats in this area, two full-length samples of bat-SL-CoVs were procured from the viral-infected bats. These two bat SL-CoVs were obtained from the same location but during different seasons; a genomic sequence identity of 88–99% was presented among them, indicating that the bats are the natural reservoirs of these SL-CoVs and that these SL-CoVs can circulate within single colonies. Meanwhile, there was a great difference between the two viruses described in this study and the viruses described in earlier studies, especially with respect to the hypervariability of the S1 domain^30,31^. It was noted that the gene encoding the S protein showed a high degree of variability. The S protein is responsible for viral entry and is functionally divided into two domains, namely, S1 and S2. The S1 domain is involved in receptor binding, while the S2 domain is involved in cellular membrane fusion. The S1 domain can be functionally subdivided into two domains, an N-terminal domain (S1-NTD) and a C-terminal domain (S1-CTD), and both can bind to host receptors, hence functioning as RBDs^32^. ZXC21 and ZC45 showed huge diversities with the previously reported CoVs of bats associated with the S1 region, and the highest level of shared identity was only 83%. An attempt was made to perform a recombination analysis during the course of this study. In our study, no potential recombination events could be identified. This could be because the two strains originated from an unsampled SL-CoV lineage residing in a bat species that is phylogenetically closer to ZXC21 and ZC45 than all other known bat SL-CoV samples. Then, we used simplot to analyze the sequence similarity of five bat-SL-CoVs and the SARS-CoV SZ3. The Longquan-140 strain is the most homologous to ZC45 and ZXC21, the Rs3367 is the closest strain of bat origin to the human pathogenic SARS coronavirus, and SZ3 is the representative strain of civetorigin.

In this study, a suckling rat model was initially used to study the possibility of the proliferation of bat-derived CoVs in other animals. Previously, only one report had shown promising results associated with the isolation of live SL-CoVs from the fecal samples of bats with Vero E6 cells^13^. The live SL-CoV cultured in Vero E6 cells presented a typical CoV morphology and has the ability to use ACE2 from humans, civets, and Chinese horseshoe bats for cell entry^33^. An attempt to isolate the virus with Vero E6 cells was unsuccessful, which was likely due to a low viral load or a lack of compatibility with Vero E6 cells. This study found that the SL-CoVs derived from bats could replicate successfully in suckling rats, and pathological examination showed the occurrence of inflammatory reactions in the examined organs of the suckling rats. This result indicated that the virus can proliferate in rats and has the potential of cross-species transmission. When CoV particles procured from the infected brain tissues of the rats were studied by electron microscopy, the morphology of the particles was found to be identical to the typical coronavirus particles, as described in previous studies^34^. However, the typical spikes could not be visualized by electron microscopy. This observation can be partially explained by the hypothesis that the S1 and S2 domains of the S protein (which are not well-connected) were easily detached from the virion using excessive freeze-thawing or ultracentrifugation^6^. Thus, there was a loss of S1 domains, which likely occurred during the preparation of the samples for electron microscopy. Meanwhile, the infected rat tissues could react with the polyclonal antibodies associated with the ZC45 N protein, according to the results from the western blotting assay, indicating that the virus can circulate in rats. Despite the negative western blotting results in the intestinal tissues of rat and the positive results of western blotting in the brain and lung tissues, we considered that these differences may be caused by different viral loads in different tissues.

In conclusion, based on the early detection of a high carrying rate for SL-CoVs, which originated from the bats in Zhoushan, China, this study involved continuous surveillance of the SL-CoVs that originated from the bats of this region. Diverse bat SL-CoVs were identified in this region, and the SL-CoVs in this region remained stable and could be transmitted to each other. Although there were several differences between the SARS-CoVs and the bat-SL-CoVs procured from this region based on the two-full-length samples obtained in this study, especially pertaining to the S protein region, this strain could still cause infection in neonatal rats. This observation highlights the possibility of cross-species transmission of these viruses. These findings strongly suggest the need for continued surveillance of viruses originating from wild animals andpromote further research to study the possibility of cross-species transmission of these viruses.

## Materials and methods

### Ethics statement

The procedures for sampling of bats were reviewed and approved by the Administrative Committee on Animal Welfare of the Institute of Zhejiang CDC Veterinary (Laboratory Animal Care and Use Committee Authorization). All live bats were maintained and handled according to the Principles and Guidelines for Laboratory Animal Medicine (2006), Ministry of Science and Technology, China. All animal experiments were approved by the Ethics Committee of the Research Institute for Medicine, Nanjing Command. All methods were performed in accordance with the relevant guidelines and regulations (Approval number: 2015011).

### Sampling

Overall, 334 adult bats were captured live at the mountain cave with mist nets at four separate times from July 2015 to February 2017 in Zhoushan city (including Dinghai and Daishan), Zhejiang Province, China. All bats appeared healthy and had no obvious clinical signs at capture. After completion of collection from each sample site, all bats were immediately dissected, and bat details are shown in Table 1. Each sample (approximately 1 g of intestinal tissues) was immediately transferred into viral transport medium (Earle’s balanced salt solution, 0.2% sodium bicarbonate, 0.5% bovine serum albumin, 18 g/l amikacin, 200 g/l vancomycin, 160 U/l nystatin), stored in liquid nitrogen prior to transportation to the laboratory, and ultimately stored at −80 °C.

### RNA extraction and RT-PCR screening

All specimens were pooled and subjected to nested RT-PCR analysis as reported in the previous study^22^. Briefly, each intestinal sample (approximately 0.1 g) was homogenized in a glass grinder with ten volumes of SM buffer (50 mM Tris, 10 mM MgSO4, 0.1 M NaCl, pH 7.5). The homogenate was centrifuged at 12,000 g for 10 min at 4 °C, but only the supernatant was used. The supernatant of each sample was passed through 0.22 μm Pellicon II filters (Millipore, Billerica, MA) to filter out the ruptured tissues, bacteria, and other impurities. The viral RNA was extracted with a Viral RNA Mini Kit (Qiagen, Hilden, Germany) according to the manufacturer’s recommendations. RNA was eluted in 35 μl RNase-free H_2_O and stored at −80 °C. Reverse transcription was carried out using the first cDNA synthesis kit (TaKaRa, Dalian, China) according to the manufacturer’s protocol with double-distilled water (ddH_2_O) as a negative control. All samples were amplified by a nested PCR that targeted a 440-nt fragment in the gene RdRp of all known alpha and betacoronaviruses^35,36^. For the first round PCR, the 20 μl reaction mix contained 18 μl of PCR reaction solution (Takara), 10 pmol of each primer and 1 μl of the DNA template. The amplification was performed under the following conditions: 94 °C for 3 min; 40 cycles at 94 °C for 30 s, 52 °C for 30 s and 72 °C for 1 min for 40 cycles of in-house reaction; and extension at 72 °C for 10 min. For the second round PCR, the 20 μl reaction mix contained 18 μl of PCR reaction buffer, 10 pmol of each primer, and 1 μl product of the first round PCR. The amplification was performed under the following conditions: 94 °C for 3 min followed by 30 cycles consisting of 94 °C for 30 s, 52 °C for 30 s, 72 °C for 30 s, and a final extension of 72 °C for 10 min with ddH_2_O as a negative control. Positive PCR products were sequenced in both directions by an ABI 3730 DNA Analyzer (Invitrogen, Beijing, China).

### Sequencing of full-length genomes

To obtain the full genomic sequences of ZXC21 and ZC45, 19 degenerated PCR primer pairs were designed by multiple alignment of available SARS-CoV and bat SL-CoV sequences deposited in GenBank, targeting almost the full length of the genome. Primer sequences are available upon request. Sequences of 5′ and 3′ genomic ends were obtained by 5′ and 3′ RACE (Takara), respectively. PCR products with expected size were gel-purified and directly subjected to sequencing. The sequences of overlapping genomic fragments were assembled to obtain the full-length genome sequences, with each overlapping sequence longer than 600 bp.

### Phylogenetic analysis of amplicons

All 440-bp-long amplicons were aligned with their closest phylogenetic neighbors in GenBank using ClustalW v.2.0. Representatives of different species in the genera of *Alphacoronavirus* and *Betacoronavirus* as well as some unapproved species were included in the alignment. Phylogenetic trees based on nucleotide sequences were constructed using the neighbor-joining method using MEGA v.7 with the Maximum Composite Likelihood model and a bootstrap value of 1000^37^.

The aligned full sequences were initially scanned for recombination events using the Recombination Detection Program (RDP)^38^. The potential recombination events between ZXC21, ZC45, Rs3367 (KC881006), Longquan-140 (KF294457.1), and HKU3-1 (DQ022305.2), as suggested by RDP with strong *P* values (<10^−20^), were investigated further by similarity plot and bootscan analyses using SimPlot v.3.5.1^39^.

### Suckling rat infecting assay

To test the pathogenicity of the ZC45 agent, infection experiments were performed in suckling rats. 3-day-old suckling BALB/c rats (SLAC, China) were intracerebrally inoculated with 20 μl of volume grinding supernatant of ZC45 intestinal tissue. Animal housing care and all animal experiments were performed in a biosafety level 3 (BSL-3) facility and were approved by the local ethics committee. After 14 days, the brain, lungs, intestine, and liver tissues from infected rats were selected to prepare pathological sections. Briefly, the tissues were fixed in 10% (vol/vol) neutral-buffered formalin. After routine tissue processing, including dehydration by graded alcohol solutions, washing, and incubation in paraffin, 4 µm thick sections were cut and stained with hematoxylin and eosin (H&E). Approximately 2 h later, the prepared tissue sections were imaged using optical microscopy (Olympus, Japan).

TEM was utilized to obtain more detailed pathological information responsible for the major symptoms. The tissue samples were fixed in 2.5% (vol/vol) dialdehyde for 2 h, postfixed in 1% (vol/vol) osmium tetroxide for 1 h, dehydrated in graded ethanol, and embedded in Epon-812 epoxy resin. Then, 70 nm ultrathin sections were produced and quickly stained in aqueous uranyl acetate and Reynolds’ lead citrate. Finally, the generated tissue sections were examined using a JEM-1200 TEM (Jeol Ltd. Tokyo, Japan).

Quantitative RT-PCR was performed using tissue suspensions of rats positive for SL-CoV by RT-PCR. cDNA was amplified in SYBR Green I fluorescence reactions (Roche) using specific primers (5′-TGTGACAGAGCCATGCCTAA-3′ and 5′-ATCTTATTACCATCAGTTGAAAGA-3′)^12^. A plasmid with the target sequence for generating the standard curve was used. At the end of the assay, PCR products (280-bp fragment of pol) were subjected to melting curve analysis (65–95 °C, 0.1 °C/s) to confirm the specificity of the assay.

### Preparation of rabbit antiserum against two peptides

To obtain the polyclonal antibody of bat SL-CoV ZC45 N protein, two partial peptides with 15-amino acid residues of N protein were synthesized (Sangon Biotech, Shanghai, China) after a homology search according to the bioinformatics analysis and prediction of signal peptide (SignalIP-4.1), hydrophilicity and antigenicity of N protein. New Zealand White rabbits (2–2.3 kg) were injected subcutaneously using 0.6 mg of two peptides in 1 ml phosphate-buffered saline (PBS) emulsified with 1 ml Freund’s complete adjuvant (Sigma). Animals were boosted twice by the same route at 2-week intervals with approximately 0.3 mg of two peptides in 1 ml of PBS emulsified with 1 ml of Freund’s incomplete adjuvant (Sigma). One week after the last booster immunization, blood samples were collected, and sera were isolated for biological activity assays. The antibody titer was tested by indirect enzyme-linked immunosorbent assay. Preimmune rabbit serum was collected before the first injection.

### Determination of virus infectivity by western blotting assay

Western blotting was performed to characterize the antigenic reactivity of infected rat tissue with N protein antibody of bat SL-CoV-ZC45. Infected intestine, lung and brain tissue samples were homogenized and lysed in RIPA buffer supplemented with proteinase inhibitors. Equal amounts of proteins (40 μg) were loaded and separated on 8% SDS-PAGE (sodium dodecyl sulfate-polyacrylamide gel electrophoresis) gel. Following electrophoresis, the proteins were transferred onto a PVDF (polyvinylidene difluoride) membrane, blocked with 5% (w/v) milk, and incubated with primary and secondary antibodies. Blots were developed and detected by enhanced chemiluminescence (GE Healthcare, Little Chalfont, UK). Rat tissues from the control specimens and intestinal tissues from bat ZC45 were used as negative and positive controls, respectively.

### Nucleotide sequence accession numbers

All amplicon sequences and the full genomes of ZXC21 and ZC45 generated in this study have been deposited in GenBank under accession numbers MG772844 through MG772934.

## Supplementary Material

Supplementary Figure S1

Supplementary Information

## Notes

**Publisher's note:** Springer Nature remains neutral with regard to jurisdictional claims in published maps and institutional affiliations.
