# Supplementary figures and images for: Genomic characterization and infectivity of a novel SARS-like coronavirus in Chinese bats
Source: Emerg Microbes Infect. 2018 Sep 12;7(1):1–10. doi: 10.1038/s41426-018-0155-5 (PMC6135831; doi:10.1038/s41426-018-0155-5)

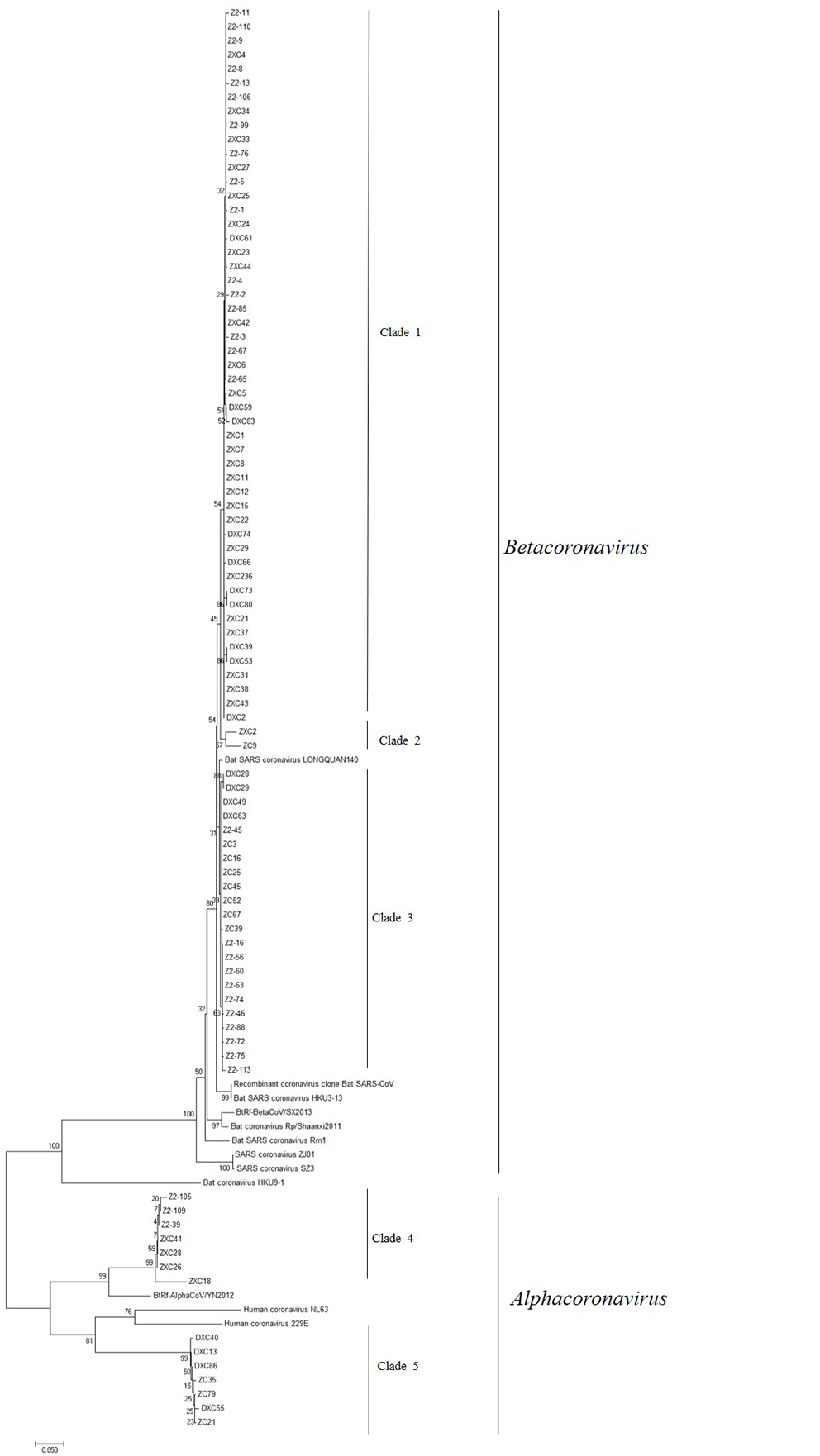

Supplement: Supplementary Figure S1 [file TEMI_A_12040062_SM0001.tif]
