# Supplementary material for: Genomic characterization and infectivity of a novel SARS-like coronavirus in Chinese bats
Source: Emerg Microbes Infect. 2018 Sep 12;7(1):1–10. doi: 10.1038/s41426-018-0155-5 (PMC6135831; doi:10.1038/s41426-018-0155-5)
Supplement: Supplementary Information [file TEMI_A_12040062_SM0002.docx]

**Supplementary Figure legend**

**Fig S1** Phylogenetic analysis of RdRp amplicons obtained in this study and representatives of species in the genera *Alphacoronavirus* and *Betacoronavirus* based on the Neighbor-Joining method. All sequences were classified into two groups: *Alphacoronavirus* comprised of 2 clades and *Betacoronavirus* comprised of 3 clades.
